# Impact of Aerated Drip Irrigation and Nitrogen Application on Soil Properties, Soil Bacterial Communities and Agronomic Traits of Cucumber in a Greenhouse System

**DOI:** 10.3390/plants12223834

**Published:** 2023-11-12

**Authors:** Zheyuan Xiao, Hongjun Lei, Yingji Lian, Zhenhua Zhang, Hongwei Pan, Chen Yin, Yecheng Dong

**Affiliations:** 1 School of Water Conservancy, North China University of Water Resources and Electric Power, Zhengzhou 450046, China; x201810102054@stu.ncwu.edu.cn (Z.X.); x20201010038@stu.ncwu.edu.cn (Y.L.); panhongwei@ncwu.edu.cn (H.P.); x202210010038@stu.ncwu.edu.cn (C.Y.); x202210010046@stu.ncwu.edu.cn (Y.D.); 2 School of Hydraulic Engineering, Ludong University, Yantai 264025, China; zhangzh71@163.com

**Keywords:** aerated drip irrigation, cucumber yield, soil properties, bacterial community, co-occurrence networks

## Abstract

Root hypoxia stress and soil nutrient turnover have been related to reduced crop productivity. Aerated drip irrigation (ADI) can effectively enhance crop productivity and yield. However, the response of the soil bacterial community to different irrigation water dissolved oxygen (DO) concentrations remains elusive due to the extreme sensitivity of microorganisms to environmental variations. We investigated the effects of aerated irrigation with different concentrations of DO on soil properties and agronomic performance of cucumber, as well as the contribution of the bacterial community. We performed experiments on cucumber cultivation in Shouguang, China, including different irrigation methods (ADI: O2–10 and O3–20 mg L^−1^, non-aerated groundwater: O1–5 mg L^−1^) and nitrogen (N) application rates: 240 and 360 kg N ha^−1^. ADI (particularly O2) significantly improved soil properties, root growth, cucumber yields, and irrigation water use efficiency (IWUE), and appropriate DO concentrations reduced N fertilizer application and increased crop yields. Furthermore, these changes were associated with bacterial community diversity, aerobic bacteria abundance, and consolidated bacterial population stability within the network module. Environmental factors such as soil respiration rate (Rs), DO, and NO_3_^−^-N have significant effects on bacterial communities. The FAPROTAX results demonstrated enhanced nitrification (*Nitrospira*) and aerobic nitrite oxidation by soil bacteria under ADI, promoting the accumulation of effective soil N and improved soil fertility and crop yield. Appropriate DO concentration is conducive to the involvement of soil bacterial communities in regulating soil properties and cucumber growth performance, which are vital for the sustainable development of facility agriculture.

## 1. Introduction

Facility agriculture is a modern agricultural method for the efficient production of plants and animals under relatively controlled environmental conditions. It has become one of the pillars of modern agriculture, gradually occupying an important position in the global food system due to the increase in population and rising demand for agricultural products [1,2]. Soil is an important natural resource for agricultural production. Crops and soil are interconnected; subsequently, they exert mutual effects on one another for effectiveness of their productivity [3]. This is because soil gives support in terms of moisture, nutrients, and anchorage to crops to grow effectively on the one hand, and on the other, crops provide protective cover for soil, suppress soil erosion as well as help to maintain soil nutrients through litter accumulation and subsequent decay [4,5,6]. The quality of soil for agricultural production depends on its sustainable supply of plant nutrients through litter accumulation and subsequent decay [6,7]. However, land productivity has been significantly hampered by groundwater contamination, fertility imbalance, and secondary salinization due to considerable changes in the soil environment; these changes are attributed to long-term cultivation, highly intensive management, and water, heat, and air disorders [8,9]. These factors increase the susceptibility of the root system of facility crops to hypoxia. In addition, soil permeability and oxygen content in the root zone of the crops are further reduced by traditional subsurface drip irrigation patterns [10,11]. Soil hypoxia is known to restrict the aerobic respiration of plant roots, and numerous metal ions accumulate in plants, hindering the growth and development of the root system and delaying the absorption of soil nutrients, thereby reducing crop production, which has become one of the factors restricting the sustainable development of agriculture [12,13,14]. Advancements in traditional subsurface drip irrigation have led to the emergence of aerated drip irrigation (ADI). Micro-bubbles produced by an ADI generator possess a large surface area, long retention time in liquids, and can continuously deliver oxygen to the root zone through subsurface drip irrigation [15,16,17]. The application of an ADI system to deliver oxygen and N fertilizers to the root zone can improve soil aeration in the root zone, alleviate soil acidification, promote the activation of quick-acting phosphorus and potassium in the soil, and enhance soil fertility and crop yield [14,18,19,20]. The positive effects of ADI on crop roots have been associated with changes in soil nutrients (soil organic carbon, total N, total phosphorus, nitrate nitrogen, etc.) [18,21]. The decomposition rate of soil organic matter (SOM) is strongly influenced by oxygen and is two to six times faster in the presence of oxygen [22].

Soil bacteria are the primary microorganisms driving plant diversity and productivity in terrestrial ecosystems [23,24], accounting for more than 70% of microorganisms, and have been implicated in nutrient cycling and plant growth [21]. However, microorganisms are susceptible to changes in the surrounding external environment [25], such as climatic changes and irrigation measures [26,27,28]. Aerobic and anaerobic microorganisms are widely distributed in the soil. Oxygen levels increase the relative abundance and activity of aerobic and anaerobic bacteria and suppress the abundance and activity of specialized anaerobic bacteria [29,30]. The altered composition of soil bacterial communities largely affects the transformation of soil carbon, N, and phosphorus [21,31]. In addition, the symbiotic network between bacterial communities promotes ecosystem multifunctionality, including nutrient cycling, regulation of environmental factors, and soil ecosystem responses to bacterial communities [32,33]. Although crop growth and soil properties are highly dependent on bacterial abundance and activity, the role of soil bacterial communities is unclear regarding regulating cucumber agronomic traits, soil properties, and yields under different concentrations of irrigation water dissolved oxygen (DO) and N applications in clay loam soils.

Therefore, we hypothesized that 10 mg L^−1^ DO in irrigation water could be more favorable to improving agronomic traits in cucumbers by altering soil properties and soil bacterial community structure. In the present study, we (1) assessed the effects of different DO concentrations and N applications on cucumber agronomic traits and soil properties, (2) investigated the responses of soil bacterial communities, potential functions, and molecular networks to different DO concentrations, and (3) revealed the relationship between soil bacterial communities and soil properties, and cucumber performance (i.e., root morphology and yield).

## 2. Materials and Methods

### 2.1. Site Description and Experimental Design

The field experiments were conducted on cucumber (variety of “No. 3966”, *Cucumis sativus* L.) in a solar greenhouse from 23 August 2020 to 10 January 2021, in Shouguang city, Shandong Province (northern China, 36°94′ N, 118°59′ E, altitude 49.5 m). The variety is mainly used for greenhouse cultivation, with high early yields, and 2~3 crops can be planted in 1 year. The study area belongs to a warm temperate continental monsoon climate, with a long-term annual mean air temperature of 12.7 °C, an annual mean precipitation of 594 mm, 2549 annual sunshine hours, and a 195 d frost-free period. The solar greenhouse covered an area of 950 m^2^ (95 m long × 10 m wide). As is common for the area, the greenhouse was configured east–west to maximize solar radiation and naturally cooled with roof vents. It is equipped with a meteorological observation station inside the greenhouse, which eliminates any interference from natural rainfall. The soil type is clay loam at 0–60 cm soil depths, with an average pH of 8.20, SOC of 15.8 g kg^−1^, total N of 1.22 g kg^−1^, total P of 1.08 g kg^−1^, and AK of 34.32 g kg^−1^. The average bulk density is 1.46 g cm^−3^, and the field capacity is 26.07%. The sand (0.02–2 mm), silt (0.002–0.02 mm), and clay (<0.002 mm) fractions of the soil were approximately 32.36%, 29.51%, and 38.13%, respectively. The experiment was set up with two types of irrigation and fertilizer application methods (ADI: 10 mg L^−1^ (O2), 20 mg L^−1^ (O3) irrigation water dissolved oxygen), and non-aeration groundwater treatment (O1, 5 mg L^−1^ dissolved oxygen). Two doses of N fertilizer were applied, including conventional N fertilization (N2, 360 kg ha^−1^), and 2/3 of conventional N fertilizer (N1, 240 kg N ha^−1^). There were six treatments, and each treatment had three replicates (Table 1). Aerated water was generated by a nano bubble generator for ADI; the equipment consisted of a circulating aeration unit, a ratio adjustment unit, a drip irrigation pressure stabilization unit, and a fiber optic oxygen spectrometer. The water supply pressure at the inlet of the pipeline was 0.1 MPa. The ADI system is irrigated when the appropriate dissolved oxygen (DO) value is reached.

Sources of N, P, and K fertilizers applied in the present study were urea (N ≥ 46% by weight), calcium superphosphate (P_2_O_5_ ≥ 12%), and potassium sulfate (K_2_O ≥ 52%), respectively. Calcium superphosphate and potassium sulfate were broadcasted at 150 kg P_2_O_5_ ha^−1^ and 200 kg K_2_O h^−1^ as the basal fertilizers one day before transplanting for the cucumber experiments; 1/3 of the total N was supplied as the basal fertilizer in each N treatment plot; the remaining N fertilizer was supplied in four equal amounts at the 36th, 52nd, 67th, and 83rd days after transplanting through a drip irrigation tube. Irrigation was performed when the E_pan_ reached 20 ± 2 mm. The irrigation was defined as the cumulative evaporation measured by an evaporation pan (20 cm in diameter and 9 cm in depth). The evaporation was measured at 8:00 a.m. every day. Each plot was equipped with a flowmeter and control valve to control the water volume. The total irrigation amount for the crop growing season was 175 mm. 

**Figure 1:**
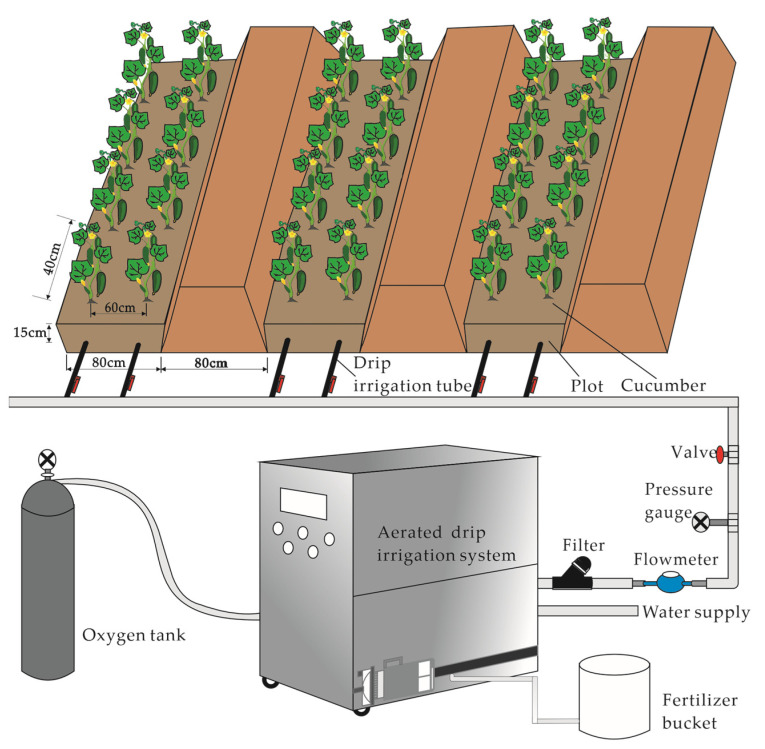
Diagram of layout of the cucumber plantation and ADI system.

The experimental layout of the ADI system and planting pattern is depicted in Figure 1. The field experiment was arranged as a split plot; each plot was 8.5 m long and 1.6 m wide, with 40 cm between plants within rows. Forty-two cucumber plants were transplanted in it. The soil surface was covered by a polyethylene film, and plastic film was placed between the plots to prevent lateral water leakage. A subsurface drip irrigation belt (16 mm in diameter, 20 cm between drips) was laid on each cultivation plot, and the buried depth was 15 cm. The waterflow of the drips was 2.2 L h^−1^. Other agronomic management includes flower spotting, mantling, pruning, and old leaf removal, with local production practices uniform.

### 2.2. Cucumber Agronomic Traits

At the fruit maturity stage, three plants were selected from each plot for destructive sampling. The stems, leaves, and fruits were weighed fresh and placed in an oven to dry to a constant weight, and then weighed. The whole root was dug out in a 40 cm × 40 cm square area, taking the selected plant as the center. The roots were washed with tap water, and the root samples were scanned with an Epson Expression 1600 Pro (Epson, Co., Ltd., Magano-ken, Japan) scanner to obtain a grayscale TIF image. The images were analyzed with a WinRHIZO software (version 5.0; Regent Instrument Inc., Quebec City, QC, Canada) image processing system to obtain the total root length (TRL), total root area (TRA), and total root volume (TRV). Five plants of cucumber were marked to determine yield in each plot, and each treatment was replicated three times. As cucumbers are picked in batches, crop yield is the sum from the first picking to the end in three replications. IWUE = Y/I, where IWUE is the irrigation water use efficiency for cucumber (kg m^−3^), Y is the yield (kg), and I is the irrigation amount (m^3^).

### 2.3. Soil Sampling

The rhizosphere soils were collected at a depth of 0–30 cm during the late maturity period of cucumber in December 2020. The samples were collected following an “S”-shaped method. For each plot, 18 samples were collected (3 replicates per treatment). Five soil samples were mixed together and divided into two parts for each plot; one was sieved through a 2 mm sieve to measure the soil chemical properties, while the other was delivered on dry ice to a sequencing company (Shanghai Personal bio Technology Co., Ltd., Shanghai, China) for DNA extraction and 16S rRNA amplicon sequencing.

### 2.4. Soil Physicochemical Properties

Soil pH was measured using a pH meter (soil to water ratio of 1:2.5). Soil NH_4_^+^–N and NO_3_^−^-N were extracted using 2 M KCl and measured by a UV-VIS spectrophotometer (Shanghai Spectrum Instruments Co., Ltd., Shanghai, China) based on indophenol blue colorimetry and dual wavelength colorimetry, respectively (Qian et al., 2022 [34]). Soil temperature was measured using a geothermometer at 20 cm depth, installed 5 cm in the plant stalk’s radial direction. Soil water dissolved oxygen content (DO) was measured using a fiber-optic oxygen meter (OXY4-mini, Presens Corp., Regensburg, Germany) with the FireSting O_2_ connected to an oxygen-sensitive probe (Robust Oxygen Miniprobe, Regensburg, Germany), the DO probe in the soil 5 cm laterally and 10 cm vertically from the plant stalk. Soil respiration rate (Rs) was measured using soil respiration measurement systems (ADC LCi-SD, Delta-T Crop., Cambridge, UK); the soil respiration chamber base was buried into the soil surface before measurement, and the data were recorded for about 5 min when the record was stable. 

### 2.5. Soil DNA Extraction, PCR Amplification, and 16S rRNA Gene Sequencing

The genomic DNA was extracted with the OMEGA M5635-02 kit for soil (MP Biomedicals, Santa Ana, CA, USA) according to the manufacturer’s instructions. DNA extraction quality was determined 1.2% by agarose gel electrophoresis, and the concentration of DNA was detected using Nanodrop 2000 (Thermo Fisher Scientific Inc., Wilmington, DE, USA). The 16S rRNA amplicons were amplified by primer pairs 338F/806R (338F: ACTCCTACGGGAGGCAGCA, 806R: TCGGACTACHVGGGTWTCTAAT) targeting the V3-V4 hypervariable regions of 16S rRNA genes [35]. DNA fragments were sequenced using the Illumina MiSeq sequencing platform. Double-end sequencing was performed on the community DNA fragments using the Illumina MiSeq platform. Library and sample segmentation by index and barcode information was performed, and barcode sequences were removed. The read sequences were compared with the Silva database to detect chimeric sequences, and the final chimeric sequences were removed to obtain the final valid data. Validated sequences were generated using QIIME2 software (version 2019.4 https://qiime2.org (accessed on 15 March 2023)), and sequences were quality filtered, denoised, merged, and removed from chimeras based on dada2 [36]. 

### 2.6. Statistical Analysis

We measured significant differences in soil physicochemical properties and plant agronomic traits among treatments using analyses of variance (ANOVA), and the means were compared by Duncan’s multiple range tests at a significant level of *p* ≤ 0.05 with the statistical software SPSS 22.0 (IBM, Chicago, IL, USA). The QIIME2 platform was used to evaluate bacterial diversity indexes (Chao1, Pielou_-_e, and Shannon). Non-metric dimensional scaling (NMDS) was performed using Bray–Curtis distances, and Anosim was used to test intergroup differences. FAPROTAX (functional annotation of prokaryotic taxa) was used to annotate amplicon sequence variants (ASVs) to obtain functional groups. Co-occurrence networks were analyzed and plotted using R software and Gephi (version 0.10.1), respectively. The key categories of the network were determined from the intra-module connectivity (Zi) and inter-module connectivity (Pi) values [37] and plotted using the R software. Other figures were constructed using the graphing software Origin 2022 (Origin Lab Corp., Northampton, MA, USA), CorelDRAW 2020 (Corel Corp. Ottawa, ON, Canada), the OmicStudio tools platform (https://www.omicstudio.cn/tool, accessed on 15 March 2023), and the Tutools platform (https://www.cloudtutu.com, accessed on 14 March 2023).

## 3. Results

### 3.1. Soil Physicochemical Properties

**Figure 2:**
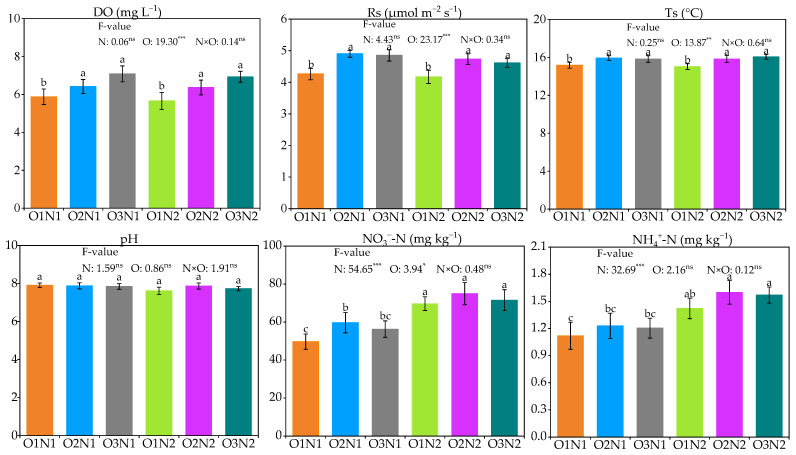
Effect of soil physicochemical properties under different treatments. The different letters indicate significant differences at 0.05. N and O represent the N application rate and irrigation method, respectively. N × O represents the effect of the interaction between the N application rate and the irrigation method. O1, O2, and O3 represent the 5, 10, and 20 mg L^−1^ irrigation water dissolved oxygen, respectively. N1 and N2 represent the 240 and 360 kg ha^−1^ N application rates, respectively. *, **, and *** identify significant differences at *p* < 0.05, *p* < 0.01, and *p* < 0.001, respectively, and ns identifies no significant.

The effect of soil physicochemical properties under different treatments is shown in Figure 2. Compared to the O1 treatment, O2 and O3 treatments significantly improved soil DO, Rs, and Ts, which increased by 15.0–27.0%, 14.0–15.2%, and 4.4–5.1% (240 kg N ha^−1^), and 12.5–22.5%, 10.4–13.4%, and 5.3–6.9% (360 kg N ha^−1^), respectively (*p* < 0.05), at 240 and 360 kg ha^−1^ N fertilizer application rates. Notably, the soil DO, Rs, and Ts were not significantly different between the two rates.

Soil pH did not vary under different treatments. Soil NO_3_^−^-N and NH_4_^+^-N content increased initially, decreased with oxygen levels, and increased with the N fertilizer application rate. The soil NO_3_^−^-N content with O2N1 was 20.1% higher than that of the O1N1 treatment (*p* < 0.05); in contrast, the differences in the soil NH_4_^+^-N content at different irrigation water DO appear under the N2 level. In addition, when the irrigation water DO was 5, 10, and 20 mg L^−1^, the average soil NO_3_^−^-N and NH_4_^+^-N content under the N2 level was 31.0% and 29.3% higher than that of the N1 treatment, respectively (*p* < 0.05).

### 3.2. Cucumber Root Growth, Biomass, Yield, and IWUE

**Figure 3:**
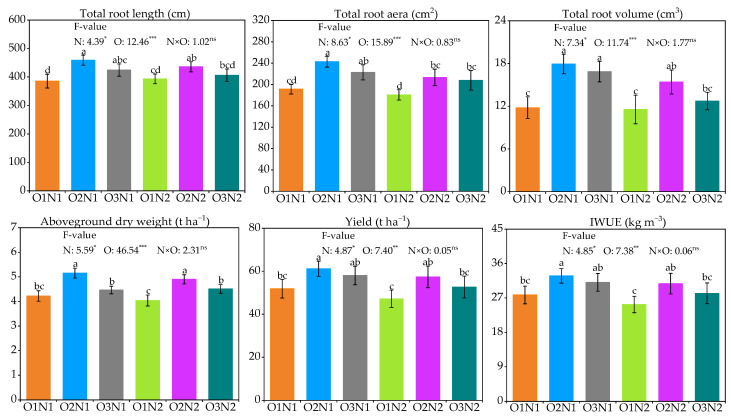
Effect of cucumber agronomic traits under different treatments; *, **, and *** identify significant differences at *p* < 0.05, *p* < 0.01, and *p* < 0.001, respectively, and ns identifies no significant difference; The different letters indicate significant differences at 0.05.

The effects of different treatments on cucumber agronomic traits are shown in Figure 3. Under two N application rates, the ADI treatments significantly increased root growth, especially with the O2 treatment. Compared with the O1 treatment, the O2 treatment significantly enhanced TRL, TRA, and TRV values by 10.8−19.1%, 18.1−26.9%, and 33.4−52.0% (*p* < 0.05), respectively, in cucumber at the two N application rates. In addition, reducing N fertilizer application at O2 and O3 levels significantly promoted the growth of cucumber TRA and TRV without significantly affecting TRL.

Aboveground dry weight (ADW), yield, and IWUE of cucumber increased, followed by a decrease with increasing oxygen levels, reaching a maximum in the O2 treatment (Figure 3). Cucumber ADW, yield, and IWUE under O2 conditions displayed average improvement of 21.7%, 19.8%, and 19.8% at two N application rates, respectively, compared with the O1 treatment. Moreover, no significant alteration was detected in different agronomic traits of cucumbers under different N fertilizer application rates.

### 3.3. Soil Bacterial Community Diversity and Composition

**Figure 4:**
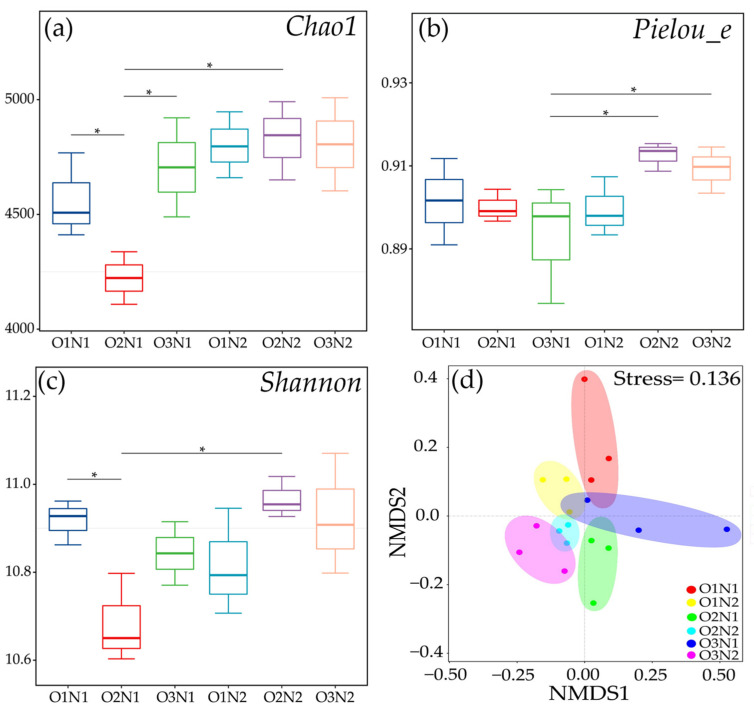
The diversity of bacterial communities in the cucumber rhizosphere different treatments. (**a**–**c**) are the Chao1, Pielou_e, and Shannon indexes of the bacterial community, respectively. (**d**) is the non-metric multi-dimensional scaling (NMDS) plot from Bray–Curtis distances of bacterial communities under different treatments; * identify significant differences at *p* < 0.05.

We conducted paired-end sequencing of community DNA fragments using the Illumina MiSeq platform. The 16S rRNA gene was quality filtered to obtain 961,144 high-quality sequence reads. Finally, 37,009 ASVs were obtained based on 97% similarity. ADI significantly affected the Chao1 and Shannon indexes of the rhizosphere soil bacteria with an insignificant effect on the Pielou_e index (Figure 4). Compared with the O1N1 treatment, the O2N1 treatment resulted in significantly reduced Chao1 and Shannon indexes (*p* < 0.05), with no significant differences in the Pielou_e index. In addition, the N2 treatment resulted in significantly greater Chao1, Shannon, and Pielou_e index values than the N1 treatment under ADI. The NMDS plot indicated distinct separation between bacterial communities of two irrigation methods (ADI (O2, O3), non-aeration (O1)), and N fertilizer application (N1, N2) treatments (except O3N1, *p* = 0.001), with significant differences under treatment (O1N1 vs. O2N1, O1N2 vs. O3N2, and O3N1 vs. O3N2) (ANOSIM, Table 2).

### 3.4. Composition and Functional Forecast of Soil Bacterial Community

**Figure 5:**
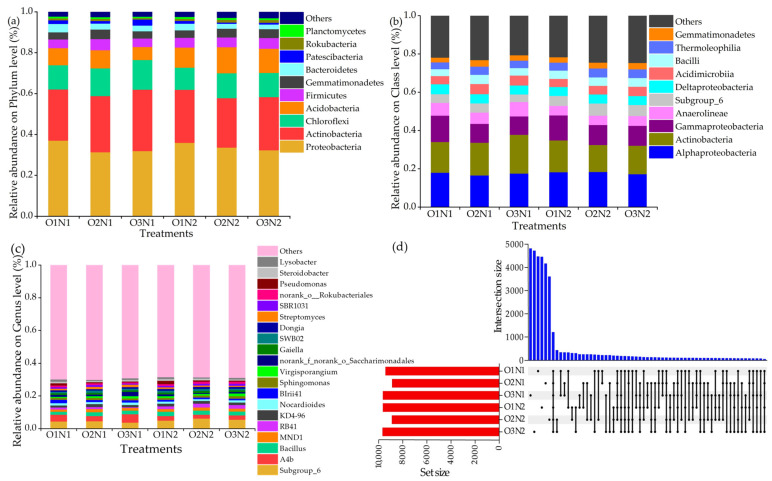
Composition of soil bacterial community at the phylum (**a**), class (**b**), genus (**c**) classification levels and Upset diagrams (**d**) under different treatments.

We analyzed the relative abundances of soil bacteria in the root zone of cucumber at the phylum (a), class (b), and genus (c) taxonomic levels under different treatments (Figure 5). At the phylum level (Figure 5a), the top five (relative abundance > 4%) relative abundances of *Proteobacteria*, *Actinobacteria*, *Chloroflexi*, *Acidobacteria*, and *Firmicutes* ranged from 31.46–37.16%, 24.16–30.06%, 10.80–14.53%, 6.37–12.78%, and 4.14–5.47%, respectively. Under two N applications, although the ADI treatment increased the relative abundance of *Chloroflexi*, it decreased the relative abundance of *Proteobacteria*. *Acidobacteria* showed an increasing trend in O1, O2, and O3 (25.04%, 27.50%, and 30.06%, respectively) with the N1 level. The dominant bacterial taxa at the class level were *Alphaproteobacteria*, *Actinobacteria*, *Gammaproteobacteria*, *Anaerolineae*, *Subgroup_6*, and *Deltaproteobacteria* (Figure 5b). We further revealed that at the two N application rates, the relative abundances of *Gammaproteobacteria* were higher in the O1 treatment than in the ADI treatment. At the genus level (Figure 5c), the dominant taxa were *subgroup_6* (3.84–6.27%), *A4b* (2.43–5.12%), *Bacillus* (1.77–2.49%), *MND1* (1.48–2.01%), *RB41* (0.44–2.35%), and *KD4–96* (1.16–1.73%). The relative abundances of *Subgroup_6* and *Bacillus* under two N applications varied among treatments and increased, followed by a decrease with the DO concentration. ADI increased by 14.17% and 17.63% compared to the non-aerated treatment. The Upset diagram demonstrates unique ASV_S_ shared under different treatments (Figure 5d). A total of 1195 shared bacterial ASV_S_ were present under the total treatments. The number of unique ASV_S_ in the O1N1 and O1N2 treatments were 4458 and 4443, respectively; in contrast, the O2N1, O3N1, O2N2, and O3N2 treatments revealed 4154, 4805, 3595, and 4707 ASVs, respectively. In addition, the number of unique ASV_S_ per treatment decreased with the DO concentration (O1–O3).

**Figure 6:**
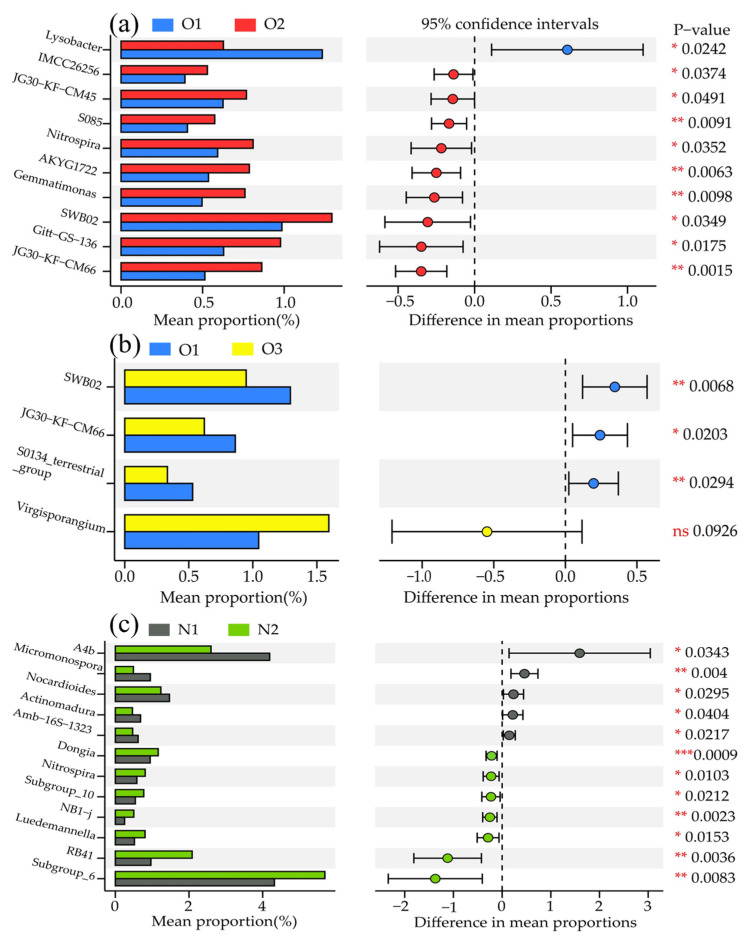
The top 50 significant differences classification levels of bacterial genus based on the STUDENT’s *t*-test under different treatments. (**a**–**c**) indicate the difference species between O1 vs O2, O1 vs O3, and N1 vs N2 treatments, respectively. *, **, and *** identify significant differences at *p* < 0.05, *p* < 0.01, and *p* < 0.001, respectively, and ns identifies no significant difference.

We further investigated the significant differences in the top 50 taxonomic levels of bacterial genus classification levels based on STUDENT’s *t*-test under different treatments (Figure 6). The mean proportions of *IMCC26256*, *JG30−KF−CM45*, *S085*, *Nitrospira*, *AKYG1722*, *Gemmatimonas*, *SWB02*, *Gitt−GS−136*, and *JG30−KF−CM66* in the O2 treatments were significantly higher than those in the O1 treatments (Figure 6a). In contrast, compared with the O1 treatment, the mean proportions of *SWB02*, *JG30−KF−CM66*, and *S0134_terrestrial_group* were significantly lower in the O3 treatment (Figure 6b). Furthermore, 12 genera were significantly different between the N1 and N2 treatments (*A4b*, *Micromonospora*, *Nocardioides*, *Actinomadura*, *Amb−16S−1323*, *Dongia*, *Nitrospira*, *Subgroup_10*, *NB1−j*, *Luedemannella*, *RB41*, and *Subgroup_6*). 

**Figure 7:**
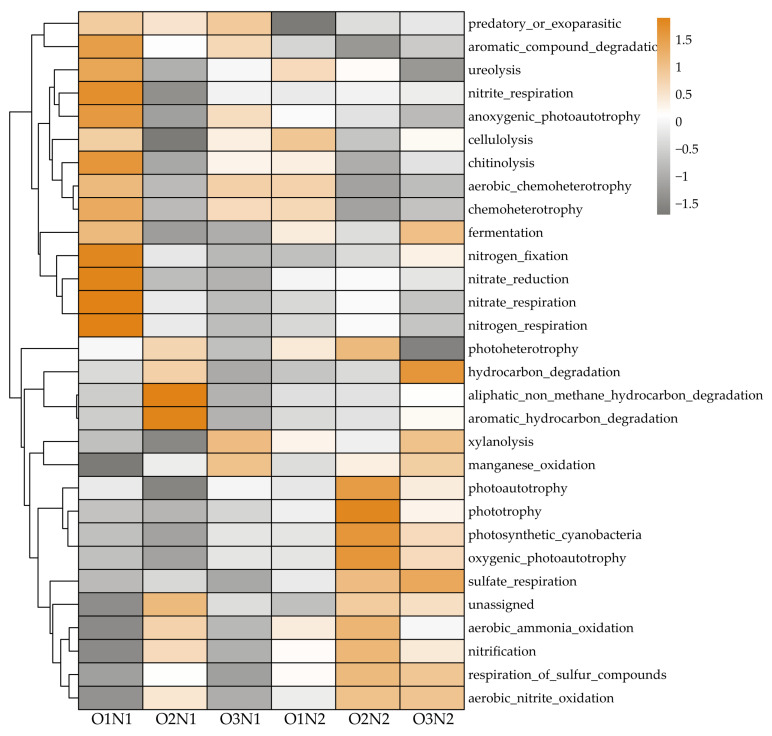
Functions of bacterial community predicted by FAPROTAX under different treatments.

The top 30 bacterial community functions identified by FAPROTAX based on ASVs are presented in Figure 7. Dominant functional groups included chemoheterotrophs (15.38%), aerobic chemoheterotrophs (14.08%), nitrification bacteria (2.55%), aerobic-ammonia-oxidation bacteria (1.94%), aromatic-compound-degradation bacteria (1.29%), and nitrate-reduction bacteria (0.98%). Overall, under two N applications, bacteria capable of nitrification, aerobic-ammonia-oxidation, aerobic-nitrite-oxidation, hydrocarbon degradation, etc., were more abundantly noticed in ADI (O2 and O3) treatment compared with the O1 treatment. In contrast, fewer groups with capacities of anoxygenic-photoautotroph, nitrate-respiration, nitrite-respiration, etc., were observed. In addition, under the O2 treatment, N2 promoted more abundant groups capable of ureolysis than N1.

### 3.5. Co-Occurrence Network Analysis of Bacterial Community 

**Figure 8:**
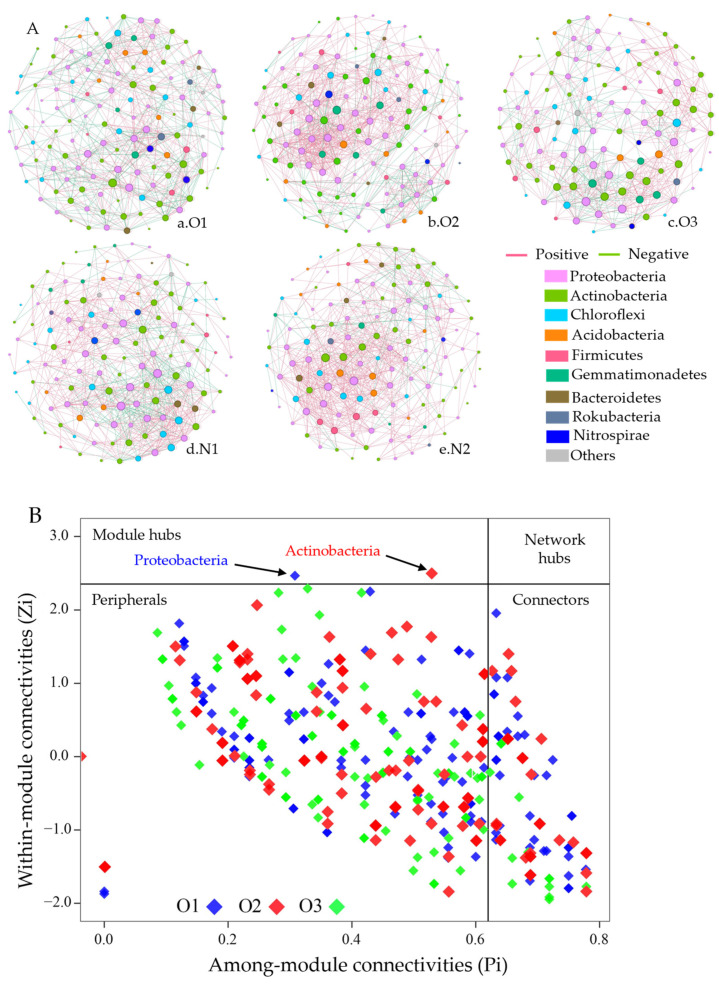
(**A**) Network co-occurrence analysis of bacterial communities from different treatments (Spearman r > 0.70 and *p* < 0.05). A node represents an ASV based on 16S rRNA, and pink (green) lines represent a positive (negative) correlation. (**B**) Zi-Pi diagram illustrating the distribution of ASVs based on topological roles. Module hubs were defined with Z > 2.5, *p* < 0.62 and connectors were defined with *p* > 0.62, Z < 2.5.3.6. Grey Correlation Analysis.

Next, the effects of different DO concentrations and N fertilizers on bacterial co-occurrence patterns were assessed. Co-occurrence networks were established based on the correlation matrix construction (Figure 8A, Table 3). Each treatment had a similarly sized network (121–140 nodes, 790–1041 edges; Table 3). Co-occurrence network features, clustering coefficient, and image density demonstrated decreasing and increasing trends with DO in the irrigation water (O1–O3), and the lowest value was present in the O2 treatment. In contrast, bacterial average degree, modularity, and P/N increased first and subsequently decreased with DO in the irrigation water. The average path length and modularity decreased with an increase in N applications, whereas the clustering coefficient, average degree, image density, and P/N increased. Moreover, networks with higher modularity displayed denser connections between nodes within modules and sparser connections between nodes in different modules. Potential keystone taxa were identified by the Zi-Pi value of each ASV (Figure 8B). The O1 network had 1 module-hub-affiliated *Proteobacteria* and 41 connectors, and 1 module hub affiliated with *Actinobacteria* and 33 connectors affiliated to *Proteobacteria*, *Actinobacteria*, *Firmicutes*, and *Acidobacteria* in the O2 network. The O3 network consisted of 19 connectors affiliated with five phyla and no module hub.

### 3.6. Relationships between the Bacterial Community, Soil Physicochemical Properties, and Cucumber Agronomic Traits

**Figure 9:**
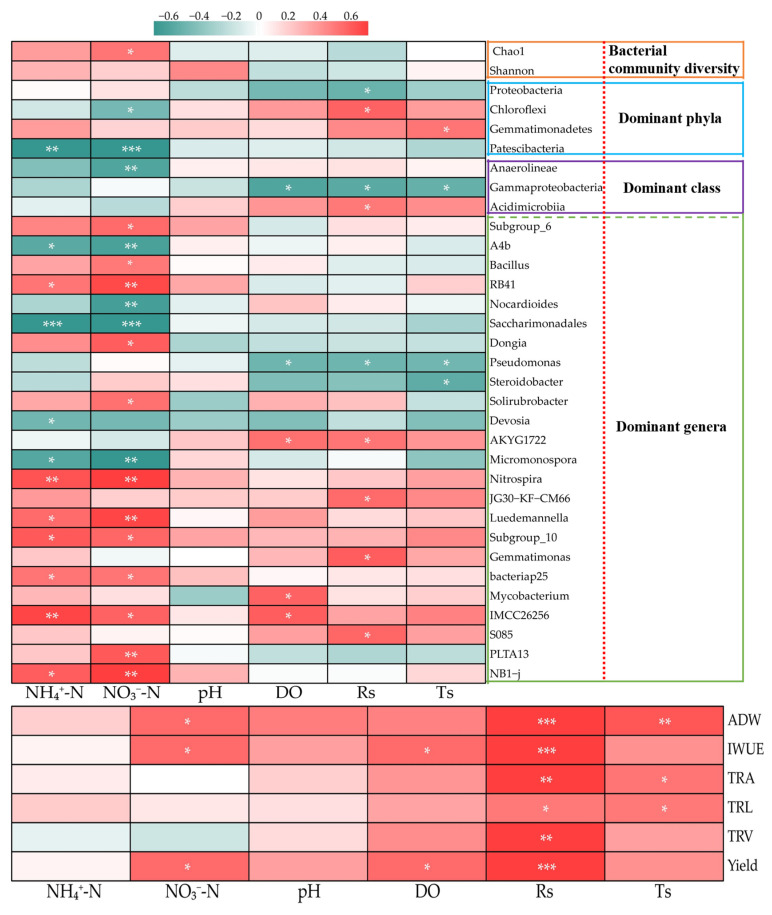
Relationships between the bacterial community, soil physicochemical parameter, and cucumber agronomic traits. *, **, and *** identify significant differences at *p* < 0.05, *p* < 0.01, and *p* < 0.001, respectively. DO, dissolved oxygen; RS, soil respiration rate; Ts, soil temperature; ADW, aboveground dry weight; TRL, total root length; TRA, total root area; TRV, total root volume.

Relationships between the bacterial community, soil physicochemical properties, and cucumber agronomic traits are depicted in Figure 9. Cucumber root growth, biomass, and yield were closely related to edaphic physicochemical properties and were impacted by soil bacterial diversity and community structure. Cucumber yield was significantly and positively correlated with soil DO, R_S_, NO_3_^−^-N, root morphology, ADW, and IWUE, which were significantly and positively associated with R_S_. We further assessed the relationships between soil physicochemical factors, bacterial community characteristics, and diversity. The Chao1 index of the bacterial community was inversely related (*p* < 0.05) to the NO_3_^−^-N of the soil. At the phylum and class levels, *Proteobacteria* and *Gammaproteobacteria* showed significantly negative correlations with R_S_; *Chloroflexi* and *Acidimicrobiia* were significantly and positively correlated with R_S_; *Patescibacteria* were significantly and negatively correlated with NO_3_^−^-N and NH_4_^+^-N; and *Gammaproteobacteria* were significantly and negatively correlated with DO, R_S_, and T_S_. Similarly, the majority of the dominant bacterial genera (relative abundance > 0.1%) showed significant correlations with soil physicochemical factors. For instance, *Nitrospira*, *Luedemannella*, and *Subgroup_10* was positively correlated with NO_3_^−^-N and NH_4_^+^-N, whereas *norank_o_Saccharimonadales* and *Micromonospora* showed negative correlations with these factors. Pseudomonas was negatively correlated with DO and R_S_. In addition, soil pH was not significantly correlated with soil physicochemical factors.

The Mantel test was conducted to quantify the relationship between soil bacterial community structure and soil physicochemical properties and identify the key environmental factors affecting changes in the bacterial community structure (Table 4). The results indicated that soil DO, Rs, and NO_3_^−^-N were the primary drivers influencing the changes in the bacterial community structure.

## 4. Discussion

### 4.1. Effects of ADI on Bacterial Community Diversity and Structure

Soil bacterial community diversity is representative of terrestrial biodiversity and is critical for regulating terrestrial biogeochemical cycles and ecosystem functioning [38]. Soil aeration to the crop root zone is known to influence the abundance and diversity of soil bacterial communities [18,34,39,40]. However, the effects of soil aeration on the abundance and diversity of bacterial communities vary depending on climatic conditions, soil type, aeration equipment, field management, and other factors. For instance, Li et al. reported significantly higher abundance and diversity of bacterial communities in aerated soils than in non-aerated soils [39]. In contrast, Zhou et al. reported lower soil bacterial abundance and diversity in soil irrigated with nano-bubble aeration than in non-aerated irrigation tomato and sugarcane root zone soils [18,40]. In addition, ADI has been reported to increase the fungal community diversity significantly but not the bacterial community diversity [21]. Our results revealed that the abundance and diversity of soil bacteria decreased, followed by an increase in the DO concentration of irrigation water at a significant level (Figure 4a,c). This phenomenon could be ascribed to higher levels of DO concentration (20 mg/L) that may directly enhance the colonization and activity of aerobic bacteria. However, microbial diversity did not continue to decline with decreasing DO concentration and increased under hypoxic levels, which was confirmed in a related study [41]. Soil NO_3_^−^-N and NH_4_^+^-N contents were significantly higher, and bacterial abundance and diversity were significantly lower at 10 mg/L DO compared to the non-aerated treatment (5 mg/L) (Figure 2). These results are consistent with those of previous studies that show that micro-nano bubble irrigation decreased bacterial diversity by increasing the content of effective nutrients and SOM [18]. The soil bacterial abundance and diversity were significantly higher at 360 kg N ha^−1^ application than at the 240 kg N ha^−1^ application at 10 mg/L DO (Figure 4a,c). Thus, the application of 360 kg ha^−1^ N fertilizer stimulated soil decomposition, accelerated N cycling and transformation, and strongly affected bacterial communities than the application of 240 kg ha^−1^ N fertilizer under the O2 treatment.

Soil oxygen concentration impacts the structure of the soil microbial community and nutrient cycling and transformation [34,42], Agricultural management practices to improve soil aeration have recently been increasingly applied. These include straw returning [43], soil aeration [44], organic fertilizer application [45], and biochar [46], Changing soil aeration could affect the components of the soil bacterial community. We found that the ADI increased the relative abundance of *Chloroflexi* and decreased that of *Proteobacteria* under two N application levels (Figure 5a). This phenomenon could be attributed to the oxygen-rich environment created by the aeration of the soil throughout the reproductive period. The soil bacteria were determined after fruit picking, during which prolonged aeration accelerated nutrient cycling, and nutrients readily decomposed in a good soil environment are efficiently absorbed and utilized by plants, thereby reducing the use of the nutrient substrate. Thus, ADI reduced the relative abundance of *Proteobacteria*. This result corroborates with that of previous studies [39,47] and the study by Šibanc et al., which reported a high abundance of *Proteobacteria* in soils with higher CO_2_ concentrations than in soils with lower CO_2_ concentrations [48]. In addition, compared to the non-aerated treatment, the relative abundance of *Firmicutes* and *Gemmatimonadetes* was higher at 10 mg L^−1^ DO. An increase in the abundance of *Gemmatimonadetes* and *Firmicutes* has been reported to accelerate carbon decomposition in an aerobic environment [49,50]. *Firmicutes* is a nutritive bacterium that converts cellulose and hemicellulose into smaller polysaccharides, thereby making it extremely resistant [39]. At the class level, *Gammaproteobacteria* exhibited a higher abundance under hypoxia (Figure 5b), as confirmed by Wu et al. [51]. In addition, *Gammaproteobacteria* contains several plant pathogens, such as Pseudomonas syringae pv. actinidiae (kiwifruit Psa outbreak) and Xylella fastidiosa [39]. In this study, we showed that the relative abundance of *Gammaproteobacteria* could be reduced under ADI, which inhibited soil diseases to certain degrees and enhanced soil health. At the genus level, the relative abundance of *Bacillus* increased, followed by a decrease with DO concentration, with a 17.63% increase under the O2 treatment compared to the O1 treatment (Figure 5c). *Bacillus* is capable of NO_3_^−^-N transformation activities under aerobic conditions [52]. In addition, O2 and N2 treatments significantly increased the relative abundance of *Nitrospirae* (Figure 6a,c) compared with the control and further promoted the uptake and utilization of soil N by plants by oxidizing soil nitrite to nitrate [40].

### 4.2. Effect of ADI on the Soil Microbial Functionality and Co-Occurrence Networks of Bacterial Community

Soil microbial communities have been widely used to predict phylogenetic traits [18,45,53]. Soil functions and variations in soil microbial community diversity and composition are strongly related to terrestrial ecosystems, especially soil carbon and N cycling [18,21]. Our FAPROTAX results demonstrated that ADI has more groups capable of nitrification, aerobic-ammonia/nitrite-oxidation, hydrocarbon degradation, and aromatic hydrocarbon degradation compared with non-aerated treatment (O1–5 mg L^−1^, Figure 7). The ADI enhanced the abundance of aerobic microorganisms and reduced the number of anaerobic microorganisms, creating an aerobic environment that promoted the proliferation of bacterial populations associated with N and carbon cycling, stimulated soil nitrification, and promoted the accumulation of effective nitrogen in the soil, thereby improving soil fertility and crop yields. In contrast, the high oxygen content accelerated water evaporation and heat loss, consequently reducing bacterial activity and the conversion of organic matter [54]. Moreover, microbial groups capable of fermentation, nitrate respiration, and nitrate reduction were active under non-aerated treatment. In addition, a higher number of photoheterotrophic groups accumulated under the O2 treatment than under the O1 treatment. These microbes utilize photo energy or oxidized organic carbon compounds to generate the chemical energy to fix inorganic carbon in the environment, thus, promoting the transition of the soil carbon pool from a stable state to an unstable state and improving the validity of the soil carbon stock [50,55,56].

We next used microbial molecular co-occurrence networks to study population interconnections within soil microbial communities [21]. Aerated irrigation changed the microbial co-occurrence pattern and increased the complexity of the network (Figure 8, Table 3). Microbial diversity has been known to be related to the complexity of the network [57]. Compared with the non-aerated and 360 kg N ha^−1^ treatments, the ADI and 240 kg N ha^−1^ treatments had lower network clustering coefficient modules and higher modularity values, with fewer links external to the modules, whereas stability is obtained by inhibiting external disturbances to the modules [58]. In addition, network aggregation is known to maintain the stability of microbial networks, and stable networks increase the resistance of bacterial communities to microenvironmental perturbations and external environmental stresses caused by network buffering [59,60,61]. In addition, species with the same ecological niche share mutualistic (positive) or competitive (negative) relationships under different environmental conditions [62,63]. The ADI and 360 kg N ha^−1^ treatments depicted higher positive correlations compared to the non-aerated and 360 kg N ha^−1^ treatment, especially with the O2 treatment, which is in agreement with the previous research, and indicates that a strong positive correlation reflects more active cooperation between the bacterial populations.

### 4.3. ADI Improved Soil Properties and Boosted Cucumber Performance by Changing the Bacterial Community

Soil bacterial communities have important implications in soil physiological and biochemical processes. They directly or indirectly influence soil organic matter decomposition and nutrient cycling by participating in the interactions between plant roots and soil properties, thereby regulating plant performance [64,65]. For instance, soil respiration releases CO_2_ into the atmosphere while consuming oxygen, and the resulting gas content gradient drives air diffusion [66]. Soil respiration is affected by the interaction of soil properties and soil bacterial communities, resulting in altered soil respiration [67]. Increased root length, surface area, and activity are related to higher area available for nutrient uptake from the soil. We demonstrated that ADI significantly enhanced Rs and root growth morphology, thereby contributing to increased yield. In addition, the Mantel test confirmed soil respiration as the most critical factor affecting microbial species composition (Table 4). A significant positive correlation was observed between soil respiration and root morphology and yield, and a significant negative correlation was reported with *Gammaproteobacteria* (Figure 9). *Gammaproteobacteria*, such as *Xanthomonas*, are plant pathogens, whose abundance reduced under ADI. DO is one of the most influential factors regulating the structure of bacterial communities as well as their diversity and abundance [68]. For instance, Qian et al. reported that good soil aeration conditions directly affected soil bacterial community composition and diversity and the root activity and metabolic activity of soil bacterial communities [34]. Plant roots can indirectly affect the growth and structure of soil microbial communities by releasing rhizosphere secretions [58]. The Mantel test demonstrated that soil DO is the second essential factor after Rs that affects microbial species composition (Table 4). Overall, aerated irrigation improved soil aeration to stimulate the proliferation of bacterial populations associated with soil N and carbon cycling and increased soil fertility, consequently promoting root growth and increasing cucumber yields, especially with 10 mg L^−1^ irrigation water DO concentration. Therefore, an appropriate concentration of DO in irrigation water is critical to improving soil health and plant growth and development. Well-developed crops affect both fruit yield and quality and are highly susceptible to disturbances in the inter-root environment. Because the soil microbial community is highly complex, the interactions and associations among its internal populations contribute to terrestrial ecosystem services. The mechanism of crop yield and quality improvement under ADI needs to be further investigated using macro-genomics, transcriptomics, and other methods, with the double objective of soil carbon sequestration and emission reduction.

## 5. Conclusions

ADI significantly improved soil properties, root growth, cucumber yields, and IWUE. In addition, it improved the appropriate DO concentration conducive to reducing the amount of N fertilizer required for crop yields, with the highest yield recorded in the O2N1 treatment (10 mg L^−1^, 240 kg N ha^−1^). Soil nutrient releasability was enhanced with 10 mg L^−1^ DO and the activity of aerobic bacteria, resulting in reduced energy consumption. Alterations in soil properties and agronomic traits in cucumbers are intricately related to changes in the soil bacterial community diversity and composition and predicted functions and symbiotic networks. Compared with the non-aerated treatment, the O2 treatment increased the network complexity of soil bacteria, with enhanced cooperation observed among the microbial network populations. Moreover, the O2 treatment boosted the soil N cycle by increasing nitrifying bacteria (*Nitrospira*) abundance and accelerating the positive feedback between microorganisms and soil nutrients. The Mantel test confirmed soil Rs, DO, and NO_3_^−^-N as major environmental factors influencing the bacterial communities. The present study was limited to the sequencing of 16S rRNA gene amplicons performed for a single crop and soil type; we plan to study the macro-genomics, transcriptomics, and other methods to identify effective methods of aerated irrigation to increase the yield and improve the quality with different crops and soil types.

## Figures and Tables

**Table 1:** Experimental treatments.

Treatments	N Fertilizer Application Rate (kg ha^−1^)	Irrigation Water Dissolved Oxygen (mg L^−1^)
O1N1	240	5
O2N1	240	10
O3N1	240	20
O1N2	360	5
O2N2	360	10
O3N2	360	20

**Table 2:** ANOSIM analysis of bacterial community under different treatments.

Group	R-Value	*p*-Value
O1N1 vs. O2N1	0.6295	0.026
O1N1 vs. O3N1	0.4424	0.105
O2N1 vs. O3N1	0.0740	0.776
O1N2 vs. O2N2	0.3704	0.112
O1N2 vs. O3N2	0.5828	0.041
O2N2 vs. O3N2	0.2222	0.280
O1N1 vs. O1N2	0.2963	0.084
O2N1 vs. O2N2	0.4526	0.103
O3N1 vs. O3N2	0.6034	0.032

**Table 3:** Topological features of the bacterial community co-occurrence networks under different treatments.

	O1	O2	O3	N1	N2
Nodes	121	140	134	135	131
Edges	897	1041	790	928	905
Average path length	3.16	2.94	3.14	2.93	2.77
Clustering coefficient	0.623	0.606	0.621	0.587	0.602
Average degree	12.81	15.54	13.06	13.75	13.82
Modularity	0.512	0.581	0.575	0.476	0.436
Image density	0.117	0.092	0.109	0.103	0.106
Positive links/Negative Links (P/N)	0.82	1.10	0.91	0.94	1.21

**Table 4:** Significance test of relationship between bacterial communities and environmental factors based on the Mantel Test.

Environmental Factor	R-Value	*p*-Value
pH	0.1099	0.266
DO	0.3105	0.007 **
Rs	0.3520	0.002 **
T_S_	0.1748	0.089
NO_3_^−^-N	0.2767	0.019 *
NH_4_^+^-N	−0.0225	0.503

Note: DO, dissolved oxygen; Rs, soil respiration rate; Ts, soil temperature; NO_3_^−^-N, nitrate nitrogen; NH_4_^+^-N, ammonium nitrogen; * and ** identify significant differences at *p* < 0.05 and *p* < 0.01, respectively.

## Data Availability

Data are contained within the article.
